# Flavonoids in Medicine and Food Homology Substances: Structure–Activity Relationship, Application Challenges, and Cutting-Edge Technological Breakthroughs

**DOI:** 10.3390/foods15040658

**Published:** 2026-02-11

**Authors:** Xuejiao Wang, Ying Li, Xiaodan Zhao, Daqi Fu

**Affiliations:** 1 School of Food and Health, Beijing Technology and Business University, Beijing 100048, China; 18533515435@163.com (X.W.); l13716504289@163.com (Y.L.); 2 College of Food Science and Nutritional Engineering, China Agricultural University, Beijing 100083, China; daqifu@cau.edu.cn

**Keywords:** medicine and food homology substances, flavonoids, structure–activity relationship, bioavailability, nanodelivery, synthetic biology

## Abstract

Flavonoids, as natural and safe bioactive compounds, demonstrate significant potential in antioxidant, anti-inflammatory, neuroprotective, and antitumor activities. Medicine and food homology substances constitute a vast treasure trove of flavonoids, characterized by high activity and high content. Their biological effects are closely linked to chemical features like hydroxyl group position, substituent type, and glycosylation degree. However, in practical applications, flavonoids in medicine and food homology substances still face bottlenecks, such as difficult separation and purification, challenging quality control, poor solubility, and low bioavailability. Current strategies include advanced extraction techniques (e.g., ultrasound/microwave-assisted, supercritical CO_2_). Quality control is achieved through establishing GAP bases, integrating data on the origins of Medicine and food homology substances, employing UHPLC-MS, and constructing fingerprint spectra. Enhancing solubility through structural modifications such as glycosylation. Utilizing nanodelivery systems such as lipid nanoparticles, polymeric nanoparticles, and microencapsulation technology to enhance bioavailability. Future research on flavonoids in medicine and food homology substances will integrate artificial intelligence (for activity prediction and formulation optimization), synthetic biology (for targeted flavonoid synthesis), and materials science (for designing novel delivery materials), advancing their applications in precision nutrition and personalized medicine. Provide a reference for fundamental research and applied development of flavonoids in medicine and food homology substances.

## 1. Introduction

Medicine and food homology substances refer to those traditionally consumed as food, listed in the Pharmacopoeia of the People’s Republic of China, with safety assessments revealing no food safety concerns, and complying with relevant laws and regulations concerning the conservation of traditional Chinese medicinal materials, wildlife protection, and ecological preservation [1]. China began compiling and promulgating the List of Substances that are Both Foodstuffs and Traditional Chinese Medicinal Materials in 2002. The list has been supplemented since then and now contains 106 substances. Nowadays, the National Health Commission of the People’s Republic of China has explicitly endorsed the advancement of a research and development platform for medicine and food homology substances, and the formulation of quality standards [2]. This marks the development and application of medicine and food homology substances and related industries, which are becoming integral components of the food and traditional Chinese medicinal materials industries.

Flavonoids have a variety of biological activities, including antioxidant, anti-inflammatory, antiviral, and immunomodulatory effects. They may help prevent diseases such as cancer, coronary heart disease, and stroke. Research also indicates they may possess potential hypoglycemic effects and exhibit anti-aging properties. Now regarded as a key ingredient in various nutritional, pharmaceutical, and cosmetic applications [3]. Medicine and food homology substances form a vast repository of flavonoids. Given their inherent properties, incorporating flavonoid-rich medicine and food homology substances into daily diets and food development ingredients represents a prudent approach to disease prevention and health promotion.

But their practical application remains challenging. For instance, flavonoids generally exhibit low bioavailability, while their content and composition within medicine and food homology substances are significantly influenced by factors such as variety, origin, harvesting, and processing. This leads to difficulties in standardizing product quality, constituting major bottlenecks in translating these substances into practical applications.

This paper provides a systematic review of the primary types, biological activities, traditional applications, modern functions, structure–activity relationships, research bottlenecks, and countermeasures concerning flavonoids within medicine and food homology substances. It aims to furnish a reference framework for both fundamental theoretical research and applied development of flavonoids within such substances.

## 2. Methods

### 2.1. Search Strategies

This review’s search scope encompasses the following electronic databases: PubMed, Web of Science, and China National Knowledge Infrastructure (CNKI). The search language was restricted to Chinese and English, with no date limitations.

The search focused on “flavonoids in medicine and food homology substances” as the core theme, employing a combination of free-text and subject terms. Keywords for both Chinese and English searches were “medicine and food homology substances” and “flavonoids”. These terms were respectively combined with keywords such as “structure–activity relationship”, “bioavailability”, “nanodelivery”, and “synthetic biology”. To minimise omissions, we conducted retrospective manual screening of the references cited in the included studies.

### 2.2. Eligibility Criteria

#### 2.2.1. Inclusion Criteria

Included studies must meet the following criteria: (a) Research subjects shall be sourced from resources explicitly recognised by authoritative Chinese institutions within the “medicine and food homology substances” catalogue. (b) Research content shall focus on flavonoids, encompassing bioactive mechanisms, structure–activity relationships between chemical structure and activity, bioavailability challenges and enhancement strategies (particularly nanodelivery), and novel production methods based on synthetic biology. (c) Literature types must comprise original research papers and systematic reviews. (d) Publications are issued in both Chinese and English to ensure the accuracy and reliability of research methodologies, results data, and interpretative conclusions.

#### 2.2.2. Exclusion Criteria

**Figure 1:**
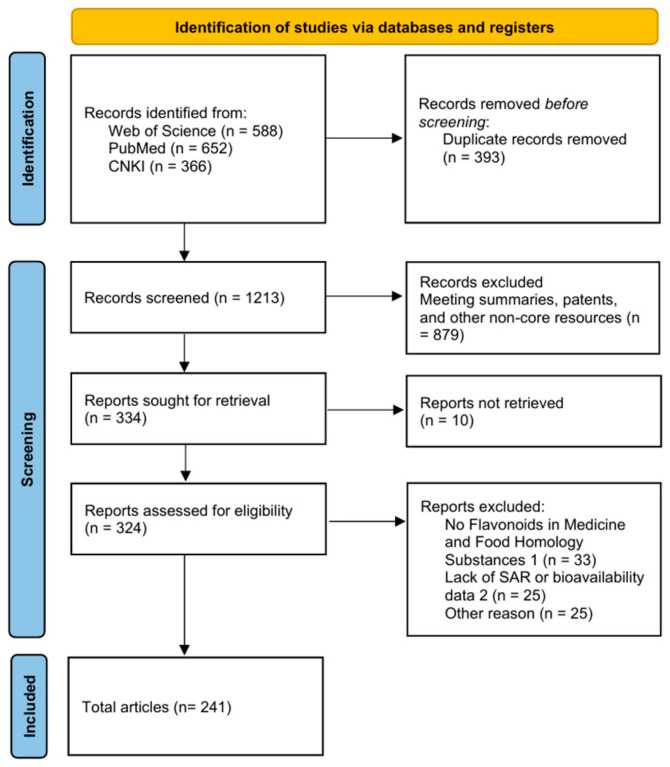
PRISMA (Preferred Reporting Items for Systematic Reviews and Meta-Analyses) flow diagram for studies retrieved through the searching and selection process.

The following studies were excluded: (a) those focusing on plants with solely medicinal value and no traditional edible history, or on common foods with no documented medicinal use; (b) articles mentioning flavonoids only as a by-product rather than the primary research focus; (c) non-core research resources (e.g., conference abstracts or patents), unless containing irreplaceable and highly relevant core data (no such instances were identified in this study); and (d) duplicate entries across databases (where a single study appears in multiple databases, only one complete record is retained). Paper screening was conducted based on the PRISMA guidelines, as illustrated in the flow diagram (Figure 1).

## 3. Structural Classification and Resource Distribution of Flavonoids in Medicine and Food Homology Substances

**Figure 2:**
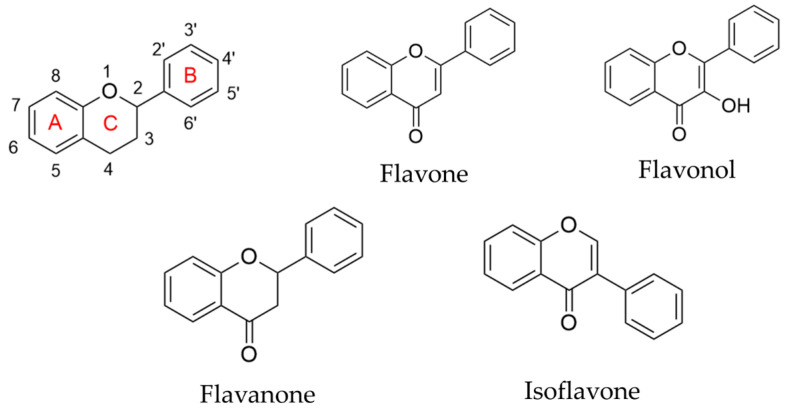
Molecular structure of flavonoids.

Flavonoids are a class of natural phenolic compounds characterized by a C6–C3–C6 carbon skeleton, consisting of two aromatic rings (A and B) linked by a three-carbon bridge that typically forms a heterocyclic C ring [4]. Based on differences in the saturation of the C-ring, the position of hydroxyl substitution, and the manner of B-ring linkage, flavonoids can be primarily classified into subclasses such as flavones, flavonols, flavanones, and isoflavones, etc. [4] (Figure 2).

The basic parent nucleus of flavones is 2-phenylchromenone, characterized by a double bond between C2 and C3 of the C ring and the absence of a hydroxyl group or other substituents at the C3 position. Flavonols possess a 3-hydroxy group on the flavone skeleton. This structural feature serves as the fundamental basis for distinguishing them from flavones and frequently influences their antioxidant activity. The C ring of flavanones lacks the C2 = C3 double bond (i.e., it is saturated between C2 and C3). This structure often renders it optically active. Isoflavones are flavonoids in which the B ring is positioned at the 3-position of the C ring, forming a unique 3-phenylchromenone basic skeleton. This structure may exhibit oestrogen-like activity.

Medicine and food homology substances are not only treasures of Chinese culinary culture but also a natural treasure trove of flavonoids. Flavonoids are widely present in medicine and food homology substances, and serve as key components responsible for their health benefits. To systematically understand their diversity and functions, we have compiled representative flavonoids from medicine and food homology substances, specifically including their compound names, subclass affiliations, primary natural sources, mechanism of action, and corresponding effects (Table 1). For the distribution of flavonoids in medicine and food homology substances, please refer to Appendix A, Table A1.

## 4. Biological Activities of Flavonoids and Their Structure–Activity Relationships

**Figure 3:**
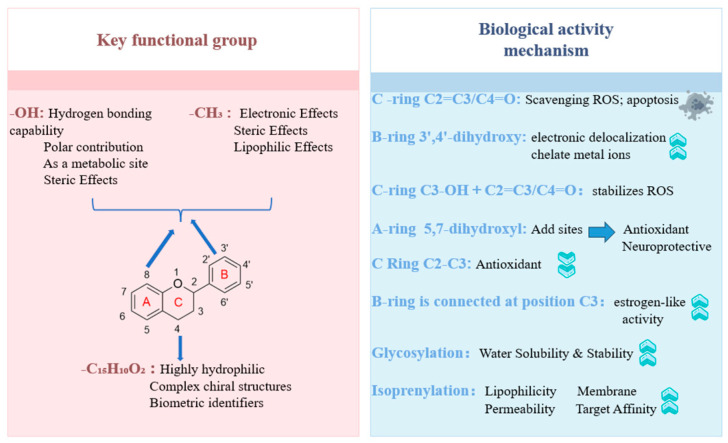
Structure–activity relationships of flavonoids: impact of functional group substitutions on core biological activities.

This chapter provides a systematic overview of the diverse biological activities and traditional and modern functionalities of flavonoids, with particular emphasis on elucidating the structure–activity relationships between chemical structures and specific biological functions. (Figure 3) The aim is to understand the mechanisms of action and structural optimisation of flavonoids within medicine and food homology substances, thereby laying the groundwork for drug design and development.

Traditional medicinal properties such as clearing heat and detoxifying, clearing the liver and improving vision, dispelling cold and alleviating pain, warming the middle burner and harmonising the stomach, strengthening the spleen and promoting digestion, regulating qi and transforming phlegm, invigorating blood circulation and dispersing blood stasis, fortifying tendons and bones, and unblocking meridians and activating collaterals [145] have been explained by modern medicine through pharmacological research. Clearing heat and detoxifying may be associated with antiviral, antioxidant, and immunomodulatory effects. Its mechanisms may involve inhibiting the release of pro-inflammatory factors such as TNF-α and IL-6; suppressing neutrophil and macrophage infiltration; reducing apoptosis; lowering ROS levels and protein expression of iNOS, COX-2, and others; and downregulating the Ras/ERK/MAPK signalling pathway to alleviate inflammatory responses and oxidative stress damage [146]. Liver-cleansing and vision-enhancing effects may be associated with antioxidant, anti-inflammatory, antiviral, and antifibrotic properties, potentially through mechanisms involving the regulation of oxidative stress, inflammation, endoplasmic reticulum stress, mitochondrial dysfunction, glucose and lipid metabolism disorders, and programmed cell death [147]. Strengthening the spleen and promoting digestion may be associated with immune regulation, anti-intestinal inflammation, modulation of gastrointestinal function and the gut microbiome, improvement of gastrointestinal barrier function, and promotion of gastric secretion [148,149,150]. Regulating qi and resolving phlegm may be associated with inflammatory responses, potentially through modulating inflammatory and immune factors to clear phlegm, alleviate airway obstruction, and enhance pulmonary function [151,152]. Promoting blood circulation and dispersing blood stasis may be associated with anti-inflammatory effects, regulation of vascular smooth muscle, and intracellular calcium signalling. Its mechanism may involve reducing atherosclerotic plaque formation, diminishing hepatic steatosis and inflammatory cell infiltration, lowering NF-κB expression, inhibiting A7r5 cell proliferation and migration, and blocking elevated intracellular calcium ion concentrations [153]. Promoting blood circulation and unblocking meridians may be associated with anti-inflammatory, antioxidant, and antibacterial activities. Its mechanisms may involve mediating PTGS2, inhibiting the synthesis of inflammatory substances, regulating inflammatory and immune responses, targeting AKT1 to exert anticancer effects, enhancing cellular stress tolerance, and participating in apoptosis [154]. Strengthening bones and muscles may be associated with anti-ageing effects, promoting bone metabolism, and repairing cartilage tissue. Its mechanism of action may involve regulating the RANK/RANKL/OPG pathway [155]. This provides objective scientific evidence for traditional experience, bridging the gap between the traditional efficacy of medicine and food homology substances and modern microscopic medicine.

### 4.1. Bioactivities and Structure–Activity Relationships of Flavones

Flavones are an important subclass of flavonoids. Examples include apigenin and luteolin. Owing to their diverse biological activities, flavones have been extensively studied and may contribute to health promotion and the prevention of chronic diseases.

Apigenin is present in the floral parts of chrysanthemums [156], and its content in the above-ground parts of *Portulaca oleracea* L. can reach 0.476–164.7 µg/g [157]. Current research indicates the potential of apigenin across multiple disease models, though the strength of evidence varies considerably with the depth of investigation. Regarding anti-inflammatory and neuroprotective effects, studies have primarily focused on animal models. In both acne-like rat models [6] and spontaneously diabetic type 2 mice (KKA^y^) [7], it has demonstrated the ability to reduce pro-inflammatory factors (such as TNF-α and IL-6), though via distinct pathways: JAK2/STAT3 and TP53, respectively. This divergence may relate to the specificity of the disease models, suggesting its anti-inflammatory effects possess multi-target characteristics, though primary targets require further validation. Regarding the inhibition of cancer cell proliferation and migration, existing conclusions are primarily based on in vitro cell experiments [8]. Although key pathways such as mTOR/STAT3 have been preliminarily identified, there is a lack of sufficient in vivo experimental evidence, and their translational potential remains unclear. In promoting hair growth, in vivo studies in mice [9] suggest it acts by activating the Wnt/β-catenin pathway, but this has not yet entered the clinical validation stage. The core effect of apigenin may be related to STAT3. By inhibiting upstream JAK2 to suppress STAT3, and STAT3 interacts with mTOR, it can alleviate inflammatory responses and inhibit proliferation and migration. Inhibiting STAT3 also helps downregulate inflammatory factors such as IL-6, improving cognitive impairment. STAT3 and Wnt/β-catenin jointly may affect cell cycle and survival.

Luteolin is abundant in the leaves of *Perilla frutescens* (L.) Britt. [158], the leaves and fruits of *Hippophae rhamnoides* L. [159], and the leaves of *Taraxacum mongolicum* Hand.-Mazz. [160]. Regarding antioxidant and cytoprotective effects, evidence primarily derives from in vitro models. In a hydrogen peroxide-induced oxidative damage model of pulmonary fibroblasts, luteolin demonstrated protective effects through mechanisms involving inhibition of lipid peroxidation and upregulation of the endogenous antioxidant enzyme system [23]. In vivo, its mechanism of action exhibits multi-pathway synergistic characteristics requiring further validation. For instance, in a D-galactose-induced brain ageing rat model, luteolin improved cognitive function through mechanisms involving SIRT1 upregulation and modulation of the AGE/RAGE pathway [24]. Regarding the potential for intervention in tumours, preliminary in vitro cellular studies indicate that luteolin inhibits the Wnt/β-catenin pathway in hepatocellular carcinoma cells (HCC), thereby suppressing their growth, proliferation, migration, and invasion [25]. However, its in vivo antitumour efficacy remains unknown, and similarly, its beneficial effects in the brain ageing model are confined to the preclinical animal study stage. The multifaceted effects of luteolin may be associated with SIRT1. By upregulating SIRT1, it modulates the downstream GLO1/AGE/RAGE pathway, thereby improving cognitive aging. Additionally, SIRT1 may exert inhibitory effects on pro-cancer pathways, such as Wnt/β-catenin.

The content of swertisin in Ziziphi Spinosae Semen can reach 1.95 ± 0.05 mg/g [161]. Regarding antioxidant effects and lifespan extension, in vitro studies and C. elegans models demonstrate that swertisin scavenges free radicals, enhances antioxidant enzyme activity (e.g., SOD, GST), and improves survival rates [162]. However, these findings only provide preliminary validation of potential mechanisms. In neuroprotection research, studies have progressed to mammalian behavioural models, yet mechanistic exploration remains limited. In animal models of despair, swertisin exhibited antidepressant-like effects, potentially mediated through monoaminergic neurotransmitter systems such as 5-HT [163]. While such studies provide in vivo pharmacodynamic evidence, proposed mechanisms remain largely indirect inferences lacking direct molecular-level substantiation. In neurodegenerative disease models, research explores mechanisms at levels closer to pathological processes. In Alzheimer’s disease models, the potential of swertisin to intervene in specific disease pathways is demonstrated by its role in alleviating cognitive impairment through modulation of the COX-2/CB1R/NF-κB pathway [164]. However, these findings remain confined to the preclinical animal experimentation stage.

Vitexin is abundant in the leaves of *Crataegus pinnatifida* Bunge [165] and in the stems and leaves of *Dendrobium officinale* Kimura & Migo [166]. In cardiovascular protection, research encompasses both in vitro and animal models, yet the elucidated mechanisms differ. In isolated animal heart models, vitexin’s improvement of cardiac function and inhibition of apoptosis are associated with regulating mitochondrial dynamics (MFN2/Drp1) and alleviating oxidative stress [34]. In diabetic rats, vitexin can ameliorate dysregulation of glucose and lipid metabolism alongside cardiac dysfunction [35]. Concerning anti-tumour and anti-ageing research, vitexin’s inhibition of breast cancer cell proliferation and migration has been validated solely in vitro through cellular experiments, with mechanisms involving regulation of targets such as MMPs/VDR [36]. In both in vivo and in vitro ageing models, vitexin’s anti-ageing effects may relate to suppression of the SASP/JAK2/STAT3 pathway [37]. However, these studies remain entirely at the basic research stage, with a considerable distance to clinical translation. The main function of vitexin may be related to the inhibition of oxidative stress and inflammation associated with aging. By regulating MFN2/Drp1 to improve mitochondrial function and reduce oxidative stress, it is precisely an important factor in delaying aging. Inhibiting the JAK2/STAT3 pathway is a key signaling axis of SASP. The down-regulation of MMPs is also regulated by SASP.

Further studies indicate that the biological activities of flavones in medicine and food homology substances are largely determined by their chemical structures, reflecting a well-defined structure–activity relationship. Key structural features include the distribution of phenolic hydroxyl groups within the molecule, the degree of saturation in the C ring, and the types of substituents present. The fundamental conjugated system formed by the C2 = C3 double bond and the C4 = O carbonyl group in the C-ring exhibits multiple biological activities. This structure can scavenge free radicals to provide antioxidant effects and induce apoptosis to exert antitumor activity [167]. The 3′,4′-dihydroxy group of the B ring (e.g., luteolin) enhances electron delocalization effects, exhibiting potent free radical scavenging and metal ion chelating capabilities [167]. This significantly boosts antioxidant, anti-inflammatory, and cardiovascular-protective activities. The 5,7-dihydroxy group in Ring A (e.g., apigenin) provides an additional active site, maintaining overall antioxidant capacity and neuroprotection [168]. Flavones commonly lack the C3-OH group, a structural feature that may reduce their polarity. Theoretically, this could facilitate penetration of cell membranes, potentially enhancing their bioavailability and tissue distribution [169]. The diverse biological activities of flavones arise from the combined structural features of the unsaturated C-ring bearing a 4-carbonyl group and the characteristic substituents on the A and B rings. However, its generally poor water solubility and the current lack of systematic clinical validation remain key bottlenecks in advancing its application towards drug development.

### 4.2. Bioactivities and Structure–Activity Relationships of Flavonols

Flavonols are an important subclass of flavonoids, represented by quercetin and kaempferol, and are widely distributed in Medicine and food homology substances. They exhibit potent antioxidant and anti-inflammatory activities, along with a broad spectrum of other pharmacological effects.

Quercetin accounts for 0.0061–0.3690% of the content in the leaves of *Crataegus pinnatifida* Bunge [170]. In terms of antitumour activity, quercetin inhibits cell proliferation and promotes apoptosis, with its mechanism potentially involving AMPK activation and COX-2 expression suppression [54]. Regarding antiviral effects, quercetin interferes with the viral replication cycle by inhibiting CDK4 [55]. Both studies are confined to in vitro cellular experiments and lack in vivo validation. In contrast, its anti-inflammatory effects have been validated through in vivo studies. In an acute pulmonary inflammation mouse model, quercetin mitigated inflammatory damage, potentially through inhibition of the PI3K/Akt/NF-κB signalling pathway [56]. The multiple biological activities of quercetin are mainly related to the regulation of metabolism and inflammation. AMPK and CDK4 are closely related to metabolism. Inhibiting PI3K/Akt/NF-κB/COX-2 is associated with the inhibition of inflammation, and it also affects cell survival and proliferation in conjunction with AMPK.

Kaempferol content in the leaves of *Hippophae rhamnoides* L. is approximately 0.332%, while that in the fruit is approximately 0.036% [171]. The content in the leaves of *Portulaca oleracea* L. is approximately 7.88 ± 0.76 mg/100 g, while that in the flowers is approximately 12.89 ± 1.31 mg/100 g [157], and it is abundant in *Eucommia folium* [172]. In the context of antitumour activity, kaempferol inhibits the proliferation and migration of HCC while inducing apoptosis, a mechanism potentially linked to the ATM/CHEK2/KNL1 cell cycle pathway [58]. However, this finding is entirely based on in vitro cellular assays (CCK-8, EDU, etc.). Regarding metabolic disorders, in leptin receptor-deficient obese mouse models, kaempferol improves body weight and alleviates insulin resistance, with its mechanism potentially involving the STING/NLRP3 pathway [59]. In neuropathic pain research, validated through both in vivo pain models and in vitro glial cell studies, the analgesic effect of kaempferol may be associated with the inhibition of the TLR4/NF-κB pathway and the regulation of glial cell polarisation [60]. This mechanism is relatively well-defined with supporting in vivo and in vitro evidence. However, there is still a lack of evidence for the effectiveness of osteoporosis treatment. Its potential action is primarily predicted through network pharmacology and has been preliminarily validated by molecular experiments demonstrating regulation of AKT1/MMP9 [61]. However, this has yet to progress to validation in cellular function or animal models. NF-κB may be an important pathway through which kaempferol exerts multiple effects. STING/NLRP3/TLR4 are its upstream pathways, AKT1 is its activator, and MMP9 is its downstream factor. Kaempferol may play a role in inhibiting tumors, improving metabolic inflammation, alleviating neuralgia, and promoting bone homeostasis by directly or indirectly affecting NF-κB.

The content of isorhamnetin in the leaves of *Hippophae rhamnoides* L. ranges from approximately 1.3 to 26.9 µg/g, in the fruit from approximately 0.160 to 9.601 µg/g, and in the seeds from 0.386 to 11.175 µg/g [173]. It is also abundant in *Illicium verum* [174]. In renal protection, in vivo studies in an acute kidney injury (AKI) mouse model demonstrate that isorhamnetin exerts a protective effect on the kidneys. Its mechanism may involve regulating the SLPI/Mincle axis to maintain macrophage phenotype, thereby inhibiting inflammation [77]. Regarding anti-fibrotic effects in the liver, isorhamnetin reduces expression of hepatic stellate cell fibrosis markers, potentially via the AKT signalling pathway [78]. This finding is based solely on in vitro HSC-T6 cell experiments, and its efficacy within the complex hepatic microenvironment remains unvalidated by in vivo studies. According to network pharmacology and cell experiments, isorhamnetin derivatives such as isorhamnetin 3-O-glycoside 7-rhamnoside and rhamnetin 3-O-rutin synergistically regulate the PI3K/AKT signaling axis through multiple targets, exerting vascular protection effects [79], without confirmation in animal models relevant to vascular function. The biological activities of isorhamnetin may be related to the regulation of PI3K/AKT signal axis. In HSC-T6 cells, it shows the activity of inhibiting PI3K/AKT pathway, reducing AKT phosphorylation, and playing an anti-fibrosis role [78]. On the contrary, in macrophage-mediated inflammation, it can activate PI3K and its downstream AKT phosphorylation [175], which can play an anti-inflammatory and vascular protection role.

Galangin content in the dried rhizomes of *Alpinia officinarum* Hance is approximately 10.58 ± 0.25 mg/g [176]. In an alcoholic liver injury model, galangin alleviated hepatic damage, oxidative stress, NLRP3 inflammatory responses, and restored the intestinal barrier in mice with high alcohol consumption [177]. This study lacked direct validation of key molecular targets or pathways. Regarding antitumour effects, galangin’s reduction in cell viability and promotion of apoptosis were confirmed solely through MTT assays and flow cytometry in vitro cell models [64]. This study did not elucidate specific mechanisms requiring further experimental validation. Regarding cardioprotection, in a DOX-induced cardiotoxicity mouse model, galangin may inhibit ferroptosis by regulating the GSTP1/JNK signalling axis [74]. Although the evidence is relatively comprehensive, clinical trial validation remains lacking.

The content of myricetin in the stems of *Portulaca oleracea* L. is approximately 10.40 µg/g, while in the leaves it is approximately 10.46 µg/g [178]. The content in *Hippophae rhamnoides* L. fruit is approximately 2994.98 ± 26.54 µg/g, while the content in leaves is approximately 4.66 ± 0.24 µg/g [72]. In Parkinson’s disease models, studies indicate that myricetin alleviates motor deficits and reduces neuronal loss in mice, potentially through activation of the Nrf2/Gpx4 signalling pathway and inhibition of ferroptosis [69]. This mechanism, associated with programmed cell death, yet its generalisability to human patients requires further validation. In pulmonary fibrosis models, myricetin reduces mouse mortality, improves lung function, and mitigates fibrosis severity. Its mechanism likely involves activating PPARγ, forming a positive feedback loop with PGC-1α to promote mitochondrial autophagy [70]. In 3×Tg mice, myricetin effectively enhanced spatial cognition, learning, and memory capacity. Its mechanisms involve anti-tau phosphorylation, synaptic protection, antioxidant effects, and improved mitochondrial function [71]. While these multiple mechanisms align with Alzheimer’s disease’s complex pathology, it remains unclear which single mechanism or combination of mechanisms specifically improves cognition.

Further research has revealed that the diverse biological activities of flavonols (such as quercetin and kaempferol) found in medicinal and edible plants are largely attributable to their characteristic chemical structures. The 3′,4′-dihydroxy structure in the B-ring stabilizes free radicals through electron delocalization, exerting potent antioxidant, anti-inflammatory, and cardiovascular-protective effects [179]. The conjugated system formed by the C2 = C3 and C4 carbonyl groups on the C-ring, together with the C3 hydroxyl group, stabilizes phenoxy radicals generated during oxidation [180]. Synergizing with the 5,7-dihydroxyl groups on the A ring enhances antioxidant and neuroprotective potential [168]. However, the presence of these highly reactive hydroxyl groups often results in poor water solubility, rapid metabolism, and low oral bioavailability, thereby limiting their clinical application [169]. Glycosylation of hydroxyl groups typically reduces in vitro antioxidant activity but may improve solubility and in vivo stability [181]. Under specific conditions, methylation or isoprenylation modifications may optimize antitumor effects by enhancing lipophilicity and membrane permeability [182]. These conclusions primarily reflect trends observed within specific research contexts, and their generalisability and practical applicability require further validation across diverse biological systems and more complex in vivo environments. The rich bioactivity of flavonols stems from the synergistic interaction between their highly conjugated molecular skeleton and characteristic hydroxyl groups. Through a profound understanding of its pharmacophore and physicochemical properties, rational structural optimisation—such as glycosylation to enhance delivery—constitutes the core strategy for overcoming clinical application bottlenecks and achieving the transition from active molecule to drug candidate.

### 4.3. Bioactivities and Structure–Activity Relationships of Flavanones

Flavanones are important members of the flavonoid family, primarily found in citrus Medicine and food homology substances. Representative compounds include naringenin, hesperetin, and eriodictyol. They have garnered significant attention due to their diverse biological activities.

Naringenin is abundant in Citri Reticulatae Pericarpium and Exocarpium Citri Grandis [93]. In the context of anti-inflammatory and anti-ageing effects, research primarily relies on cellular senescence models. Naringenin has been demonstrated to inhibit cellular senescence and inflammation, with its mechanism potentially involving suppression of the NF-κB pathway and downstream inflammatory mediators [92]. This has been validated by numerous studies, exhibiting clear in vitro pharmacological activity. However, the efficacy of this mechanism in vivo requires further validation through animal and clinical research. Concerning cardioprotection and antitumour effects, research has primarily focused on animal models. For instance, in mice with septicaemic cardiomyopathy, naringenin targets the HIF-1α pathway, thereby alleviating myocardial cell swelling, inflammatory responses, and septicaemic cardiomyocyte apoptosis [93]. While in xenograft tumour models, it induces apoptosis and autophagy via the reactive oxygen species (ROS)/AMP-activated protein kinase (AMPK) pathway, significantly inhibiting tumour growth and promoting cell death [94]. These differing mechanisms indicate a high degree of dependence on the pathological context. Regarding metabolic regulation, studies in high-fat diet rat models demonstrate that naringenin improves lipid metabolism, a mechanism that similarly involves the AMPK pathway [95].

Neohesperidin is abundant in Citri Reticulatae Pericarpium and Exocarpium Citri Grandis [105,106]. Existing research demonstrates the protective potential of neohesperidin in models of inflammation-related diseases, though its efficacy and mechanisms vary across different models. In a combined model of colorectal cancer and inflammatory bowel disease, neohesperidin effectively suppressed tumour proliferation, induced apoptosis, and inhibited angiogenesis in an in vivo mouse model induced by nitrosomethane (AOM)/sodium dextran sulphate (DSS). This mechanism may be associated with the inhibition of the NF-κB/p65/ERK/p38 MAPK pathway [100]. This finding links anti-inflammatory and anticancer mechanisms, providing valuable preclinical evidence for the prevention of colitis-associated colorectal cancer progression. In acute neurotrauma models, neohesperidin demonstrated therapeutic effects on traumatic brain injury in rats. Its mechanism involves regulating inflammatory factor gene expression (downregulating IL-6, etc.) and promoting reparative factor expression (upregulating VEGF, etc.) [102]. However, the study’s explanation of signalling pathways remains broad. In neurodegenerative disease models, neohesperidin inhibits neuroinflammation, modulates gut microbiota imbalance, alleviates motor deficits, and mitigates neuronal damage in MPTP-induced Parkinson’s disease mice. Its mechanism may involve the NF-κB/MAPK pathway [108]. However, its efficacy in humans requires validation through clinical trials. Neohesperidin may exert its various effects primarily through the inhibition of the NF-κB/MAPK pathway. This pathway mediates the release of pro-inflammatory cytokines such as IL-6 and IL-1β, thereby suppressing inflammatory responses. Additionally, it upregulates mRNA expression of VEGF, SRC, Akt1, etc., reducing neuronal damage and apoptosis.

Eriocitrin is abundant in *Mentha canadensis* L. and Citri Reticulatae Pericarpium [109,110]. In terms of anti-fibrotic and anti-inflammatory effects, eriocitrin demonstrated favourable ameliorative outcomes in both the intraperitoneal thioacetamide (TAA)-induced liver fibrosis mouse model [109] and the carotid atherosclerosis rat model [111], with mechanisms involving regulation of inflammation and oxidative stress. However, their core pathways differ, being PPARα-NLRP1/NLRC4 and Nrf2/HO-1/NF-κB respectively. Regarding anti-angiogenic and anti-tumour effects, both in vitro and in vivo studies have been conducted. Its anti-angiogenic activity is primarily demonstrated in human umbilical vein endothelial cell (HUVECs) models, where it inhibits tube formation and promotes apoptosis. The mechanism may involve multiple pathways, including MAPK/ERK and VEGF2 [110], providing preliminary cellular-level mechanistic clues for subsequent in vivo research. Regarding oesophageal carcinoma, combined KYSE30 cell experiments and nude mouse xenograft models suggest that eriocitrin may induce ferroptosis by inhibiting the STAT3/GPX4 pathway [108,112]. This mechanism is relatively novel but requires further experimental validation.

Further research reveals that the diverse biological activities of flavanones in Medicine and food homology substances are intrinsically linked to their unique chemical structure, exhibiting distinct structure–activity relationships compared to flavones and flavonols. The core structure of flavanones comprises the C2–C3 single bond and chiral center in the C ring. This configuration results in a lack of planarity and conjugated systems within the molecule, thereby diminishing its capacity to act as an electron or hydrogen donor. Consequently, in most in vitro antioxidant models based on electron transfer mechanisms, flavonones typically exhibit lower activity than flavones and flavanols possessing planar conjugated structures [183]. The antioxidant activity of flavanones primarily relies on the B-ring’s phenolic structure and the 5,7-dihydroxyl group on the A-ring. Isoprenylation at the 6,8 positions significantly enhances antioxidant activity [184]. Non-planar structures and chiral centres confer unique potential for biological interactions, such as the anti-inflammatory effect of 4′-oxy substitution on the B-ring inhibiting COX-2, iNOS, and NF-κB pathways. Furthermore, molecular flexibility and stereochemical features may enhance selective binding to relevant targets [185]. Isoprenylated flavanones exhibit enhanced lipophilicity and target affinity, leading to superior anti-inflammatory and antitumor activities [186]. Prenylation, synergistically acting with saturated C-rings and chiral centres, not only enhances lipophilicity and membrane permeability but also significantly induces tumour cell apoptosis and cell cycle arrest via stereospecific mechanisms, thereby potentially conferring antitumour activity [187]. The biological activity of flavanones results from the combined effects of their saturated C-ring skeleton, characteristic hydroxyl substituents (particularly 4′-OH on the B-ring and 7-OH on the A-ring), and isoprenylation modifications. The C2–C3 single bond and chiral centre constitute the key structural basis for understanding its relatively weak antioxidant activity, while exhibiting unique selectivity in anti-inflammatory and anti-tumour effects.

### 4.4. Bioactivities and Structure–Activity Relationships of Isoflavones

Isoflavones are a structurally unique class of compounds within the flavonoid family, primarily distributed in Medicine and food homology substances such as *Glycine max* (L.) Merr. and Puerariae Lobatae Radix. Compounds like genistein and puerarin exhibit diverse biological activities.

The genistein content in Puerariae Lobatae Radix is approximately 336 mg/kg, while that in *Glycine max* (L.) Merr. is about 30 mg/kg [188]. Research into neuroprotection and antitumour effects has primarily focused on animal models. In rats with traumatic brain injury (TBI), genistein alleviates anxiety-like behaviour and neurological deficits while significantly reducing cerebral oedema. Its mechanism of action involves inhibiting the NLRP/caspase-1 signalling pathway, thereby mitigating anxiety-like behaviour in TBI rats [128]. In syngeneic tumour-bearing mice, genistein inhibits the proliferation of anti-castration prostate cancer cells and tumour formation in vivo, acting through the inhibition of the AKR1C3 receptor [129]. In pulmonary fibrosis research, combining in vivo and in vitro models, genistein effectively prevents fibroblast activation. Its mechanism may involve regulating signalling pathways, including the UPR, EMT, and mTORC1 [130]. Regarding metabolic regulation, in a high-fat diet mouse model [131], genistein significantly suppressed weight gain, hyperglycaemia, and lipid deposition in adipose tissue and liver by modulating gluconeogenesis via miR-451. Research based on network pharmacology predictions suggests that regulating the MMP, PARP1, PLAU and PTGS2 pathways may be associated with angiogenesis [127] that require validation through cellular and animal studies.

The content of puerarin in Puerariae Lobatae Radix is approximately 1.08 ± 0.02 mg/g [189]. In mice rendered obese by a high-fat diet, puerarin effectively alleviates insulin resistance, reduces body weight, and improves glucose tolerance and insulin sensitivity. Its mechanism of action involves inhibiting the JNK and IKKβ/NF-κB pathways, thereby lowering levels of TNF-α and IL-6 [139]. In a chicken model, puerarin mitigates oxidative damage to the liver and thymus induced by oxidised soybean oil, acting through inhibition of the Nrf2/Keap1/HMGB1/TLR4/MAPK signalling pathway [140]. Regarding neurological disorders and antitumour effects, research primarily centres on animal models. In sepsis-associated encephalopathy (SAE) rats [190], puerarin effectively suppressed NLRP3/Caspase-1/GSDMD-mediated pyroptosis, thereby protecting the blood-brain barrier. In pancreatic ductal adenocarcinoma (PDAC) xenograft mice [141], puerarin effectively inhibited tumour growth and metastasis by suppressing the classical Akt/mTOR growth signalling pathway.

The content of daidzein in Puerariae Lobatae Radix is approximately 10,436.16 ± 2143.83 mg/kg [188]. Research into the biological activity of daidzein has primarily focused on animal models. Regarding neuroprotection, daidzein improves behavioural and cognitive function in chronically unpredictable mildly stressed (CUMS) mice by activating the ERβ receptor and modulating the ERK/mTOR signalling pathway [136]. In lipopolysaccharide-induced mastitis mice, daidzein effectively mitigated lipopolysaccharide-induced mammary tissue pathology by inhibiting MAPK and threonine protein kinase/NF-κB signalling pathways. This action modulated the release of interleukins (IL-6 and IL-1β) and enzymes involved in reactive oxygen and nitrogen metabolite metabolism [137].

Further research revealed that the bioactivity of isoflavones in medicine and food homology substances primarily stems from the structure where the B ring is attached at the C3 position. This configuration results in a smaller conjugated system, reducing direct antioxidant capacity but conferring significant estrogen-like activity [191]. The 4′-position hydroxyl group modulates the androgen receptor and inhibits kinase activity, effects potentially linked to antitumour, anti-inflammatory, and neuroprotective actions. However, these complex biological activities remain determined by the molecular structure as a whole, substituents at other sites, metabolic stability, and multi-target interactions [192]. The 7-hydroxy group and its glycosides (such as daidzin and puerarin) exert cardiovascular-protective activity and enhance bioavailability [193]. The diverse biological activities of isoflavones result from the combined effects of the B-ring-C3 linkage, the 4′-OH group, and the 7-substitution on the A-ring.

### 4.5. Discussion

Based on the aforementioned research findings concerning biological activity, certain correlations have been identified between the traditional therapeutic effects of medicinal and food homology substances and modern medical science. Alleviating dry eye syndrome may be regarded as one of the therapeutic effects for improving vision, with its potential mechanisms possibly involving the suppression of oxidative stress [194]. Compounds such as apigenin, erioictrin, and daidzein, which exhibit significant antioxidant and anti-inflammatory activity, may contribute to the anti-dry eye effects observed in chrysanthemum (apigenin), *Mentha canadensis* L., (erioictrin) and *Glycine max* (L.) Merr. (daidzein). (The parentheses indicate the main flavonoids contained in the plant. the same below). Alleviating atherosclerosis may be regarded as one of the therapeutic effects of ‘activating blood circulation and resolving stasis’. This may be related to mechanisms such as anti-inflammatory, reduced NF-κB expression, and diminished oxidative stress [153]. Flavonoids including luteolin, vitexin, quercetin, kaempferol, isorhamnetin, and myricetin exhibit anti-inflammatory and antioxidant activities. These compounds may help the anti-atherosclerotic effects of *Crataegus pinnatifida* Bunge (luteolin, quercetin) and *Hippophae rhamnoides* L. (vitexin, kaempferol, isorhamnetin, and myricetin). The treatment of sepsis may be regarded as one of the therapeutic effects of ‘clearing heat and detoxifying, promoting qi circulation and harmonising the stomach’. Its potential mechanisms may involve inhibiting pro-inflammatory cytokines, downregulating ROCK2 and suppressing NF-κB, alleviating oxidative stress, and mitigating liver and gastric damage [195]. Compounds such as apigenin, luteolin, and myricetin exhibit anti-inflammatory, antioxidant, and anti-apoptotic activities. These may help the treatment of sepsis within plants including *Chrysanthemum* (apigenin), *Portulaca oleracea* L. (apigenin, myricetin), *Taraxacum mongolicum* Hand.-Mazz. (luteolin), and *Perilla frutescens* (L.) Britt. (luteolin). Alleviating airway obstruction and enhancing pulmonary function may be regarded as one manifestation of the therapeutic effect known as ‘regulating qi and resolving phlegm’. The potential mechanism may involve regulating inflammatory and immune factors [151,152]. Flavonoids such as luteolin, vitexin, naringenin, neohesperidin, and eriocitrin possess anti-inflammatory and antibacterial activities, which may benefit to the effects of *Hippophae rhamnoides* L. (luteolin), *Dendrobium officinale* Kimura & Migo (vitexin), Citri Reticulatae Pericarpium (naringenin, neohesperidin, and eriocitrin), and Citri Grandis Exocarpium (naringenin, neohesperidin) to relieve airway obstruction and enhance lung function. The therapeutic effects of treating rheumatoid arthritis may be regarded as one manifestation of ‘unblocking meridians and activating collaterals’. Its mechanism may be associated with regulating inflammatory and immune responses, participating in apoptosis, and enhancing cellular stress tolerance [154]. Compounds such as genistein, puerarin, and daidzein possess anti-inflammatory, antioxidant, and anti-apoptotic activities, and may play a role in the therapeutic effects of Puerariae Lobatae Radix in treating arthritis. Maintaining bone homeostasis may be regarded as one of the therapeutic effects of ‘strengthening the bones and tendons’. Its mechanism may be related to regulating the RANK/RANKL/OPG pathway [155]. Kaempferol, possessing anti-osteoporotic activity, may contribute to *Eucommia folium*’s maintenance of bone homeostasis. With the extensive and in-depth advancement of research in this field, the modern medical mechanisms underlying traditional therapeutic effects will be subjected to more thorough and comprehensive analysis. Further functions of flavonoids within medicinal and food homology substances may be identified, with their efficacy and mechanisms receiving more robust substantiation.

Based on the preceding detailed discussion of flavonoids and their activities, their biological potency is primarily determined by the combined effects of the core skeleton and substituents. For instance, the molecular planarity and conjugated system maintained by the C2–C3 double bond (present in flavones, flavanols, and isoflavones) are typically associated with potent in vitro antioxidant and kinase inhibitory activities. Subtle structural variations between subclasses may confer unique advantages in anti-inflammatory, neuroprotective, or oestrogen-modulating effects.

Flavonoids are always coexisting with proteins, dietary fibers, and lipids in medicine and food homology substances of the same origin. These components may affect their stability and biological activity [196,197]. The existing research is basically on individual flavonoids, which neglects the influence of coexisting components. Meanwhile, most reports on the synergistic effects of flavonoids are limited to simple mixtures of a few monomers, lacking discussions in the context of real food matrices [198]. A more fundamental limitation is that the research on their mechanism of action mostly remains at the level of describing that a certain flavonoid “may affect” a certain signaling pathway, lacking studies on upstream initial molecular targets, such as receptors and kinases [199,200]. This results in a large number of conclusions only proving “correlation” rather than “causation”. Therefore, future research should be conducted in a real food environment, integrating the multi-target effects of flavonoids to prove their overall mechanism of action.

Most flavonoids have poor intestinal absorption, rapid metabolism, and low oral bioavailability. The existing data are basically based on in vitro cell experiments and animal models, and their clinical effects are still unknown. The effective concentrations in vitro may not be achievable in vivo [201]. Concurrently, variations in the composition and function of individual gut microbiota also influence flavonoid metabolism and bioactivity [202]. This leads to significant discrepancies between in vitro cell and animal models and clinical trial results across different populations. To address this issue, future research should incorporate in vitro models capable of simulating intestinal fluid shear stress and microenvironments [203], alongside next-generation pharmacokinetic-pharmacodynamic models integrating microbial functional parameters [204]. This approach will enable the simulation of population-specific differences in flavonoid metabolism. Conduct biomarker-guided precision nutrition and drug clinical trials.

## 5. Challenges and Cutting-Edge Solutions in the Application of Flavonoids from Medicine and Food Homology Substances

Transforming these medicines and food homology substances from medicinal ingredients into modern health products still faces numerous challenges. This chapter will systematically analyze the core bottlenecks constraining the application of flavonoids in Medicine and food homology substances and outline corresponding solutions. This lays the scientific foundation for systematically developing this green pharmaceutical treasure trove.

### 5.1. Complex Composition and Quality Control Challenges

The content and composition of flavonoids in medicine and food homology substances are influenced by factors such as species, origin, and harvest season. Species, organ, growing environment, and stress conditions collectively determine their content and diversity [205]. The content and types of flavonoids vary across different plant parts, such as flowers, fruits, leaves, roots, and seeds. The concentration of myricetin differs between the stems and leaves of *Portulaca oleracea* L. [178]. Galangin is primarily concentrated in the dried rhizomes of *Alpinia officinarum* Hance [176]. Factors such as light exposure, soil composition, and temperature can lead to variations in the types and content of flavonoids. For instance, flavonoid content differs among Citri Reticulatae Pericarpium from various production regions [206]. Biotic and abiotic stresses, including drought, salinity, heavy metals and mechanical injury, also exert a significant influence on flavonoid content [207].

Existing solutions include establishing Good Agricultural Practice (GAP) bases for the cultivation and breeding of Chinese medicinal herbs, implementing standardised management. For example, prioritising superior varieties of Lycium chinense Miller to yield large fruits with stable polysaccharide and flavonoid content [208]. In the cultivation of chrysanthemums, agricultural, biological and physical control methods are employed to minimise the use of chemical pesticides [209]. By integrating artificial intelligence with environmental big data analysis, establishing a mapping model between flavonoid content and environmental factors can enhance the predictability and uniformity of raw materials. Existing research has preliminarily identified key combinations of environmental factors influencing flavonoid content in economically valuable species such as *Lycium chinense* Miller through machine learning analyses (e.g., random forests, neural networks) [210]. Using gene editing technologies such as CRISPR/Cas9, perform targeted knockouts, insertions, or replacements in the genomes of medicine and food homology substances. Developed highly active, readily soluble flavonoids [211]. Researchers have successfully developed *Glycine max* (L.) Merr. varieties with several times higher isoflavone content than conventional cultivars by precisely upregulating the GmMYB14 transcription factor using CRISPR activation technology [212]. Precision regulation of endogenous metabolic networks in medicine and food homology substances through plant chassis engineering enables direct improvement of the resources themselves. It may alleviate the common bottlenecks in flavonoid production, such as low yields, slow growth, and unstable quality [213,214]. Researchers have achieved a several-fold to several-tens-fold increase in anthocyanin content by regulating the overexpression of transcription factors such as MYB and bHLH in Lycium chinense Miller, thereby synergistically activating multiple structural genes within the flavonoid synthesis pathway [215].

At the acquisition and application level, the extraction of target flavonoids is costly and prone to batch-to-batch quality inconsistencies [216]. Structurally similar flavonoid isomers require high-resolution chromatography techniques for separation. Inadequate quality control standards, coupled with cost considerations, mean many standards measure only one or two biomarkers. Evaluating just one or a few active components fails to reflect the product’s overall efficacy; single-parameter quality control struggles to comprehensively assess the product’s overall quality [217,218]. For example, the markers for Exocarpium Citri Grandis are limited to only two compounds: Naringin and Rhoifolin [219].

Ultrasonic/microwave-assisted extraction and supercritical CO_2_ extraction may be employed as environmentally sound and highly efficient extraction techniques [220]. Researchers employed microwave-assisted extraction to isolate baicalin from *Scutellaria baicalensis* Georgi [221]. In terms of quality control, using modern analytical techniques such as high-performance liquid chromatography and ultra-high-performance liquid chromatography-mass spectrometry (UHPLC-MS), establish chemical fingerprint profiles of the composition and distribution characteristics of flavonoid glycoside-aglycone communities [222]. Researchers have established a flavonoid chemical fingerprint profile for *Lonicera japonica* flowers using UHPLC-MS, revealing the compositional characteristics of its glycoside-aglycone community [223]. At the mechanism analysis level, a multidimensional network linking ‘constituents-targets-pathways-diseases’ was constructed through network pharmacology to predict and validate the synergistic regulation of key signalling pathways by multiple flavonoids. Subsequently, metabolomics analysis examined holistic shifts in endogenous metabolites, integrating chemometric methods to establish quality markers [224]. Researchers have conducted network pharmacology and metabolomics analyses of *Crataegus pinnatifida* Bunge, revealing its comprehensive mechanism of action through the synergistic regulation of multiple active components across core pathways related to lipid metabolism, inflammation, and energy [225].

### 5.2. Improving the Pharmacokinetic Properties of Flavonoids

Flavonoids derived from medicine and food homology substances exhibit exceptional biological activity in vitro but demonstrate poor pharmacokinetic properties in vivo. This substantially limits their translation into clinical efficacy. This issue is not unique to flavonoids classified as medicine and food homology substances, but rather stems from the shared structural characteristics of this class of compounds.

The main problems include: Poor water solubility impedes flavonoid absorption, resulting in low oral bioavailability [226]. Low membrane permeability results in flavonoids being actively pumped out of cells, reducing their intracellular accumulation and hindering their penetration through cell membranes to reach target sites [227]. Structure is unstable, sensitive to pH, and prone to oxidation, leading to structural changes [228]. Through gut microbiota metabolism, flavonoid glycosides undergo hydrolysis, resulting in cleavage of the C-ring structure and consequently low absorption rates [229]. Though two-phase metabolism significantly restricts the entry of numerous flavonoid aglycones into systemic circulation, their absolute bioavailability is neither uniform nor uniformly low. For instance, flavanols such as (-)-epigallocatechin (EGCG) often exhibit bioavailability below 1% [230], whereas daidzein may exceed 20% [135]. Consequently, most flavonoids demonstrate low bioavailability, though precise values depend upon both the specific subclass and individual compound.

Existing solutions include the use of nanoscale drug delivery systems (such as nanocrystals [231], polymeric nanoparticles [232], and lipid nanocarriers [233]) improves the water solubility of flavonoids, enhances stability, increases targeting efficiency, protects against enzymatic degradation, and prolongs their duration of action in vivo [233]. Researchers have successfully prepared diosmin nanocrystals (~277 nm particle size) stabilized by HPMC E15, significantly improving drug release behavior [234]. Eudragit E PO copolymer encapsulates flavonoid-enriched fractions of *Passiflora caerulea* L., enhancing antidepressant activity tenfold [235]. Nanostructured lipid carriers loaded with *Zingiber officinale* Roscoe extract enhance the stability and bioavailability of *Zingiber officinale* Roscoe flavonoids [236].

Improving flavonoids at the molecular level through structural modification and derivatization, such as methylation of hydroxyl groups and glycosylation, to enhance their activity, stability, and pharmaceutical properties [237]. Glycosylation modification introduces hydrophilic sugar moieties to enhance the water solubility of flavonoids [237]. Researchers have introduced hydrophilic glycosyl groups at the C3 position of 3-hydroxyflavone and quercetin, the C3 position of the B-ring of 3-methoxyflavone, and the C7 position of baicalein, significantly enhancing the water solubility of these four flavonoids [238]. Methylation at the C6 position of baicalein enhances its inhibitory activity against certain cancer cells (such as HepG2 liver cancer cells) to three times that of the parent compound [239].

When flavonoids are combined with enzyme inhibitors, the latter can reduce the metabolism of flavonoids, thereby increasing their blood concentration and bioavailability. Current studies have demonstrated that combining hesperetin with piperine increases hesperetin’s blood concentration by 1.5–2 times or more [240]. Quercetin enhances the bioavailability of EGCG in mice by approximately threefold through inhibition of catechol-O-methyltransferase (COMT) and multidrug resistance proteins (MRPs), while significantly reducing its methylation metabolism [241].

## 6. Conclusions

This review systematically elucidates the chemical diversity, traditional applications, and modern functions, structure–activity relationships, application bottlenecks, and emerging solutions for flavonoids derived from medicine and food homology substances. These compounds are abundant in a wide range of botanicals, have a long history of dietary and therapeutic use, and generally exhibit favorable safety profiles. They display significant bioactivities, including antioxidant, anti-inflammatory, neuroprotective, and antitumor effects, thereby exemplifying the modern relevance of the traditional “food-medicine homology” concept in integrative health science.

The chemical structure of flavonoids is closely related to their biological activity. Structural features such as hydroxyl position, substituent type, and degree of glycosylation directly determine their mechanism of action and efficacy. However, practical applications still face bottlenecks such as difficulties in extraction and purification, low quality control, poor solubility, and low bioavailability. Currently, separation and purification methods such as ultrasonic/microwave-assisted extraction and supercritical CO_2_ fluid extraction are being employed, while novel extraction and purification techniques like deep co-solvent extraction and membrane separation technology are also undergoing continuous development. Quality control is achieved through establishing GAP bases, integrating data on the origins of Medicine and food homology substances, employing UHPLC-MS, and constructing fingerprint spectra. Enhancing solubility through structural modifications such as glycosylation, improving bioavailability using nanodelivery systems, including lipid nanoparticles, polymeric nanoparticles, and microencapsulation techniques. This establishes a technological foundation for the development of functional foods and pharmaceuticals.

In the future, with the application of artificial intelligence in activity prediction and formulation design, the optimization of flavonoid biosynthetic pathways through synthetic biology, and innovations in advanced materials for delivery systems, flavonoid research in medicine and food homology substances will enter a new phase of deep multidisciplinary integration. These advancements will drive practical applications in precision nutrition and personalized medicine, while also providing solutions for developing next-generation health products and therapeutic drugs derived from natural sources. They hold broad prospects for both scientific research and market development.

## Figures and Tables

**Table 1:** Composition, sources, mechanism of action, and corresponding effects of representative flavonoids in medicine and food homology substances.

Types of Flavonoids	Representative Compounds	Number of Main Medicine and Food Homology Substances	Mechanism	Action	References
flavones	apigenin	13	Inhibit JAK2/STAT3	Inhibit pro-inflammatory factors	[5,6,7,8,9,10,11,12,13,14,15,16,17,18,19,20,21,22]
			Intervene in the Hippo pathway	Promote cell proliferation and inhibit apoptosis	
			Activate the Wnt/β-catenin	Regulate the hair growth cycle	
	luteolin	11	↑ SIRT1, modulates AGE/RAGE	Antioxidant, anti-aging	[10,11,20,21,23,24,25,26,27,28,29,30]
			↓ ICAM-1/RHOC/MMP2	Anti-inflammatory, inhibits cell migration and invasion	
			Inhibits Wnt/β-catenin activation	Suppress tumor growth, metastasis	
	chrysin	2	Inhibit HMGB1 and modulate RAGE/PI3K/AKT	Inhibit neuroinflammation	[31,32,33]
			Suppress apoptosis in the hippocampus and prefrontal cortex	brain protection	
			regulate CDK1/ULK1	Protect the nervous system	
	vitexin and its derivatives	5	↑ MFN2, inhibiting Drp1 mitochondrial recruitment	Improve mitochondrial function and reduce oxidative stress	[19,22,34,35,36,37,38,39]
			Inhibit MCF-7, MMP-2/9, ↑ VDR, TIMP-2	Inhibit the proliferation and migration of breast cancer cells	
			Inhibit SASP, JAK2, STAT3, PI3K, AKT, mTOR, SREBP-1c	Delaying aging and fibroblast senescence	
	baicalein	2	Block TLR4, MyD88, NF-κB	Anti-inflammatory	[40,41]
			Block JAK2/STAT3	Inhibit pro-inflammatory factors	
			↑ GPX4	Inhibit ferroptosis	
flavonols	quercetin and its derivatives	26	Activate Nrf2/HO-1/NQO1/γ-GCS	Antioxidant, ↑ cellular resistance	[15,17,19,22,27,28,29,38,40,42,43,44,45,46,47,48,49,50,51,52,53,54,55,56,57]
			Activate AMPK, inhibit COX-2	Inhibit tumor cell proliferation and promote apoptosis	
			Inhibit MCF-7 and HT-29 cells	Inhibit cancer cell activity	
			Inhibit CDK4 overexpression	Inhibit virus and protect cells	
			Inhibit PI3K/Akt/NF-κB	Reduce pneumonia damage	
	kaempferol	19	Regulate ATM/CHEK2/KNL1	Inhibit liver cancer cell migration and growth, induce apoptosis	[11,15,17,22,27,38,43,45,46,49,50,53,57,58,59,60,61,62,63,64,65,66,67]
			Regulate STING/NLRP3	Anti-obesity, alleviate inflammation and insulin resistance	
			Inhibit TLR4/NF-κB and regulate the M1/M2 polarization balance	Reduce neurotoxic proteins and pro-inflammatory cytokines	
			↑ AKT1, ↓ MMP9	Promotes bone formation and inhibits bone resorption	
	myricetin	10	Activate Nrf2/Gpx4	Improve neurological function	[11,18,19,27,50,57,63,67,68,69,70,71,72]
			Activate PPARγ/PGC-1α	Improve lung and cognitive function, anti-fibrotic	
	galangin	1	Alleviate liver damage, oxidation, and NLRP3 inflammation	Repair damaged intestinal barrier function	[64,73,74,75]
			Induces apoptosis in MCF-7 and LNCaP cells	Inhibit cancer cell growth	
			Regulates GSTP1/JNK	Protect the myocardium	
	isorhamnetin and its derivatives	7	Activate SLPI, ↓Mincle/Syk/NF-κB	Inhibit macrophage inflammation and protect the kidneys	[30,38,42,45,52,53,57,76,77,78,79]
			↓ COLA1, α-SMA	Inhibit core fibrotic cells	
			Regulate PI3K/AKT-eNOS	Antioxidant, protects endothelial function and blood vessels	
			Inhibit p38/JNK MAPK	Reduce liver damage and inflammation	
	rutin	19	Regulates PPARγ	Improve lipid metabolism	[13,18,20,22,28,29,38,50,51,57,80,81,82,83,84]
			↓ NF-Κb/STAT3/IL-6/TNF-α/IL-7	Inhibit inflammation and autoimmune responses	
			↓PI3K/AKT/mTOR/Wnt/β-catenin	Inhibit cell proliferation	
	hyperoside	10	Activate JAK2, STAT3, JNK, Keap1, Nrf2, HO-1	↑cells’ self-defense and repair	[13,15,27,29,38,40,43,45,47,51,62,67,85,86,87,88,89,90]
			Target NRF2/SLC7A11/GPX4	Induce cell death	
flavanones	naringenin and its derivatives	15	Inhibits NF-κB	↓IL-1β, IL-6, IL-8	[14,18,19,21,22,42,43,50,84,91,92,93,94,95,96,97,98,99]
			Inhibits HIF-1α	Reduce myocardial swelling, inflammation, and septic apoptosis	
			Mediates ROS production and activates AMPK	Inhibit tumor growth and promote cell apoptosis	
	neohesperidin	4	Inhibits NF-κB/p65/ERK/p38/MAPK	Anti-tumor, inhibits angiogenesis	[100,101,102,103,104,105,106,107,108]
			↓ IL-6, IL-1β, TNF-α; ↑ VEGF, SRC, Akt1 mRNA	Reduce neuronal cell damage and inhibit apoptosis	
	eriocitrin	2	Regulate PPARα-NLRP1, NLRC4	Inhibit fiber formation	[109,110,111,112]
			Regulate MAPK, ERK, VEGF2, PI3K, AKT, mTOR	Inhibit angiogenesis, induce apoptosis	
			Regulate Nrf2, NQO-1, HO-1, NF-κB	Anti-atherosclerosis	
			Inhibit STAT3/GPX4	↑ ROS/MDA/Fe2+, ferroptosis	
	hesperetin	5	Activate the PI3K/AKT/Nrf2	↑ cell protection and antioxidant	[20,96,97,98,106,107,113,114,115,116,117]
			Inhibit NF-κB activation	Inhibit pro-inflammatory factors	
			↑SOD/GPx/GR/GCLC/HO-1; ↓TNF-α, IL-6	Anti-inflammatory, antioxidant	
	pinostrobin	4	↓ROS, activate Nrf-2	Protect immune cells	[30,63,118,119,120,121,122,123]
			Inhibit DAAM2/Wnt/β-catenin	Anti-tumor	
			Activate STING/TBK1/IRF3	Inhibit tumor cell proliferation	
	epicatechin	10	Regulate COL1A1, FGF-2, GPX-1, MMP-1 expression, apoptosis	Inhibit oxidative damage, protect fibroblasts	[13,15,16,17,18,19,28,44,50,88,124,125]
			Regulate AKT-P53/CREB	Maintain cellular homeostasis	
isoflavones	genistein	8	Regulate MMP, PARP1, PLAU, PTGS2	Regulate angiogenesis, apoptosis, osteogenic differentiation	[11,15,28,38,52,126,127,128,129,130,131,132,133,134]
			Inhibit Nlrp/caspase-1	Improve anxiety, neurological function, brain edema	
			Inhibits AKR1C3 receptors	Inhibit cancer cell proliferation	
			Regulate UPR, EMT, mTORC1	Inhibit fibroblast activation	
			Regulate the expression of Gk and G6pc through miR-451	Inhibit gluconeogenesis	
	daidzein	5	Activate ERβ receptors and regulate ERK/mTOR	Reduce depressive and anxious behaviors, improve motor and memory functions	[15,133,134,135,136,137,138]
			↓ IL-6, IL-1β, iNOS, COX-2	Reduce pathological damage to breast tissue	
	puerarin	1	Inhibit JNK/IKKβ/NF-κB; ↓ TNF-α, IL-6	Reduce insulin resistance and weight	[139,140,141]
			Inhibit Nrf2, Keap1, HMGB1, TLR4, MAPK	Reduce oxidative damage to the liver and thymus	
			Inhibit NLRP3/Caspase-1/GSDMD	Protect the blood-brain barrier	
			Inhibit Akt/mTOR	Inhibit tumor growth and metastasis	
	calycosin	2	↑ Nrf2/SLC7A11/GPX4/GSS/GCL	Antioxidant, block tumor growth and promote its death	[142,143,144]

↑ = upregulation/promotion; ↓ = downregulation/inhibition.

## Data Availability

No new data were created or analyzed in this study.

## Abbreviations

The following abbreviations are used in this manuscript:

UHPLC-MS	ultra-high-performance liquid chromatography-mass spectrometry
GAP	Good Agricultural Practices
CNKI	China National Knowledge Infrastructure
HCC	hepatocellular carcinoma cell
AKI	acute kidney injury
AMPK	AMP-activated protein kinase
AOM	nitrosomethane
DSS	sodium dextran sulfate
TBI	traumatic brain injury
HUVECs	human umbilical vein endothelial cells
CUMS	chronic unpredictable mild stress
ROS	reactive oxygen species
EGCG	(-)-epigallocatechin
PDAC	pancreatic ductal adenocarcinoma
COMT	catechol-O-methyltransferase
MRPs	multidrug resistance proteins

## Appendix A

**Table A1:** List of medicine and food homology substances of various flavonoids.

Flavonoids	Medicine and Food Homology Substances
apigenin	Chrysanthemum, *Portulaca oleracea* L., *Perilla frutescens* (L.) Britt., *Crataegus pinnatifida* Bunge, fructus cannabis, *Lilium brownii* var. *viridulum* Baker, *Myristica fragrans* Houtt., *Cinnamomum cassia* (L.) D. Don, *Euryale ferox*, Semen Hoveniae, *Taraxacum mongolicum* Hand.-Mazz., *Mentha canadensis* L., *Dendrobium officinale* Kimura & Migo
luteolin	Coicis Semen, *Zingiber officinale* Roscoe, Chrysanthemum, *Portulaca oleracea* L., *Perilla frutescens* (L.) Britt., *Hippophae rhamnoides* L., *Prunus mume* Siebold & Zucc., Lonicerae Japonicae Flos, *Alpinia officinarum* Hance, *Taraxacum mongolicum* Hand.-Mazz., *Mentha canadensis* L.
chrysin	*Alpinia oxyphylla* Miq., Astragali Radix
vitexin and its derivatives	*Crataegus pinnatifida* Bunge, *Illicium verum*, Semen Hoveniae, *Lophatherum gracile* Brongn., *Dendrobium officinale* Kimura & Migo
baicalein	Longan Arillus, *Eucommia folium*
quercetin and its derivatives	Bitter Apricot Seed, Coicis Semen, *Amomum villosum* Lour., Semen Raphani, *Hippophae rhamnoides* L., *Alpinia oxyphylla* Miq., *Zingiber officinale* Roscoe, Mulberry Leaf, *Ecklonia kurome*, Lotus Leaf, *Ganoderma lucidum* (Curtis) P. Karst., *Illicium verum*, *Crataegus pinnatifida* Bunge, *Portulaca oleracea* L., *Prunus mume* Siebold & Zucc., *Polygonatum odoratum* (Mill.) Druce, *Lilium brownii* var. *viridulum* Baker, *Cinnamomum cassia* (L.) D. Don, *Phyllanthus emblica* L., *Houttuynia cordata* Thunb., Semen Hoveniae, *Lycium chinense* Miller, *Lophatherum gracile* Brongn., Croci Stigma, *Dendrobium officinale* Kimura & Migo, Flos Sophorae Immaturus
kaempferol	Coicis Semen, *Hippophae rhamnoides* L., *Gardenia jasminoides* J. Ellis, *Siraitia grosvenorii* (Swingle) C. Jeffrey ex Lu et Z. Y. Zhang, *Alpinia oxyphylla* Miq., *Zingiber officinale* Roscoe, *Alpinia officinarum* Hance, *Portulaca oleracea* L., Mulberry Leaf, *Ecklonia kurome*, *Ganoderma lucidum* (Curtis) P. Karst., *Eucommia folium*, *Illicium verum*, *Polygonatum odoratum* (Mill.) Druce, *Lilium brownii* var. *viridulum* Baker, *Cinnamomum cassia* (L.) D. Don, *Phyllanthus emblica* L., *Lycium chinense* Miller, *Dendrobium officinale* Kimura & Migo
myricetin	*Alpinia oxyphylla* Miq., *Zingiber officinale* Roscoe, *Portulaca oleracea* L., *Ganoderma lucidum* (Curtis) P. Karst., *Phyllanthus emblica* L., *Euryale ferox*, Hordei Fructus Germinatus, Semen Hoveniae, *Lycium chinense* Miller, *Hippophae rhamnoides* L.
galangin	*Alpinia officinarum* Hance
isorhamnetin and its derivatives	Bitter Apricot Seed, *Hippophae rhamnoides* L., *Illicium verum*, *Lycium chinense* Miller, *Alpinia officinarum* Hance, *Lophatherum gracile* Brongn., Croci Stigma
rutin	Coicis Semen, *Hippophae rhamnoides* L., *Gardenia jasminoides* J. Ellis, *Zingiber officinale* Roscoe, *Eucommia folium*, *Illicium verum*, *Crataegus pinnatifida* Bunge, *Prunus mume* Siebold & Zucc., Longan Arillus, Mulberry Leaf, *Phyllanthus emblica* L., *Citrus medica* ‘Fingered’, *Euryale ferox*, Hordei Fructus Germinatus, Lonicerae Japonicae Flos, *Houttuynia cordata* Thunb., *Lycium chinense* Miller, *Taraxacum mongolicum* Hand.-Mazz., *Dendrobium officinale* Kimura & Migo
hyperoside	*Hippophae rhamnoides* L., Longan Arillus, Lotus Leaf, *Eucommia folium*, *Illicium verum*, *Crataegus pinnatifida* Bunge, *Pseudocydonia sinensis* (Dum. Cours.) C. K. Schneid., *Lilium brownii* var. *viridulum* Baker, Lonicerae Japonicae Flos, *Houttuynia cordata* Thunb.
naringenin and its derivatives	*Ziziphus jujuba* Mill., Bitter Apricot Seed, Coicis Semen, *Hippophae rhamnoides* L., Mulberry, Citri Reticulatae Pericarpium, Exocarpium Citri Grandis, fructus cannabis, *Phyllanthus emblica* L., *Citrus medica* ‘Fingered’, *Euryale ferox*, Semen Hoveniae, *Mentha canadensis* L., *Rubus idaeus* L., *Dendrobium officinale* Kimura & Migo
neohesperidin	*Citrus medica* L., Citri Reticulatae Pericarpium, Exocarpium Citri Grandis, *Citrus aurantium* L. var. *amara* Engl.
eriocitrin	*Mentha canadensis* L., Citri Reticulatae Pericarpium
hesperetin	Mulberry, Citri Reticulatae Pericarpium, Exocarpium Citri Grandis, *Portulaca oleracea* L., *Taraxacum mongolicum* Hand.-Mazz.
pinostrobin	*Alpinia oxyphylla* Miq., honey, *Glycyrrhiza uralensis* Fisch., *Alpinia officinarum* Hance
epicatechin	*Crataegus pinnatifida* Bunge, *Amomum villosum* Lour., *Prunus mume* Siebold & Zucc., *Pseudocydonia sinensis* (Dum. Cours.) C. K. Schneid., *Lilium brownii* var. *viridulum* Baker, *Myristica fragrans* Houtt., *Cinnamomum cassia* (L.) D. Don, *Phyllanthus emblica* L., *Euryale ferox*, Semen Hoveniae
genistein	*Portulaca oleracea* L., Puerariae Lobatae Radix, *Illicium verum*, *Prunus mume* Siebold & Zucc., *Lilium brownii* var. *viridulum* Baker, Galli Gigerii Endothelium Coreneum, *Lophatherum gracile* Brongn., Sojae Semen Praeparatum
daidzein	Puerariae Lobatae Radix, *Lilium brownii* var. *viridulum* Baker, Galli Gigerii Endothelium Coreneum, Sojae Semen Praeparatum, *Cistanche deserticola* Ma
puerarin	Puerariae Lobatae Radix
calycosin	Astragali Radix, *Glycyrrhiza uralensis* Fisch.
